# Supplementary material for: Neurovascularization inhibiting dual responsive hydrogel for alleviating the progression of osteoarthritis
Source: Nat Commun. 2025 Feb 6;16:1390. doi: 10.1038/s41467-025-56727-8 (PMC11799281; doi:10.1038/s41467-025-56727-8)
Supplement: Supplementary file 1 — Supplementary Information [file 41467_2025_56727_MOESM1_ESM.pdf]

Supplementary Information

Neurovascularization inhibiting dual responsive hydrogel for alleviating the progression of osteoarthritis

This SI file includes:

Figure S1. The FTIR results of SA, SAP and OSAP, showing the synthesis of OSAP polymers.
Figure S2. The FTIR results of PEI, PCA and PPCA, showing the synthesis of PPCA polymers.
Figure S3. The characterization of BGN and BGN@Be.
Figure S4. SEM-EDS of OSPP hydrogel and OSPPB hydrogel (areas without BGN@Be distribution).
Figure S5. Photographs of RBCs after treatment with OSPPB, with the corresponding statistical results.
Figure S6. Representative fluorescence images of LIVE/DEAD staining of EPCs after OSPPB treatment.
Figure S7. CCK-8 assay of the viability of EPCs and TG cells under the stimulation of different groups.
Figure S8. H&E staining of the major organs, including heart, liver, spleen, lungs, and kidneys after OSPPB intraarticular injection.
Figure S9. The distribution of the rhodamine B-stained hydrogel in condyles.
Figure S10. Original images of the condyles used for SEM-EDS.
Table S1. Primer sequences used for qRT-PCR in the present study.

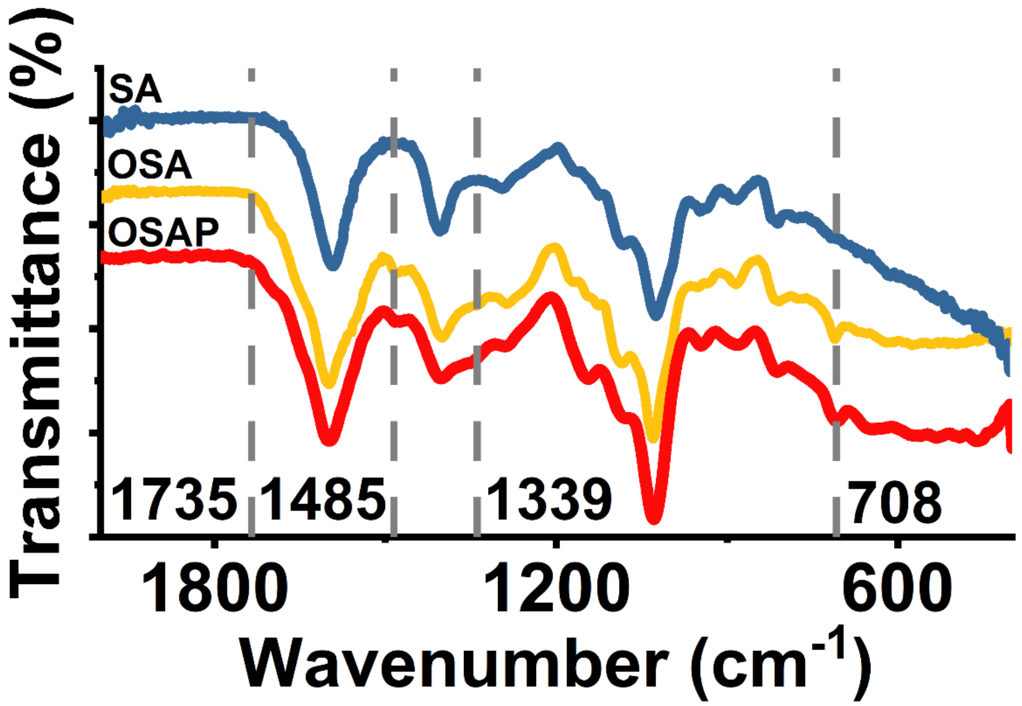

Figure S1. The FTIR results of SA, SAP and OSAP, showing the synthesis of OSAP polymers.

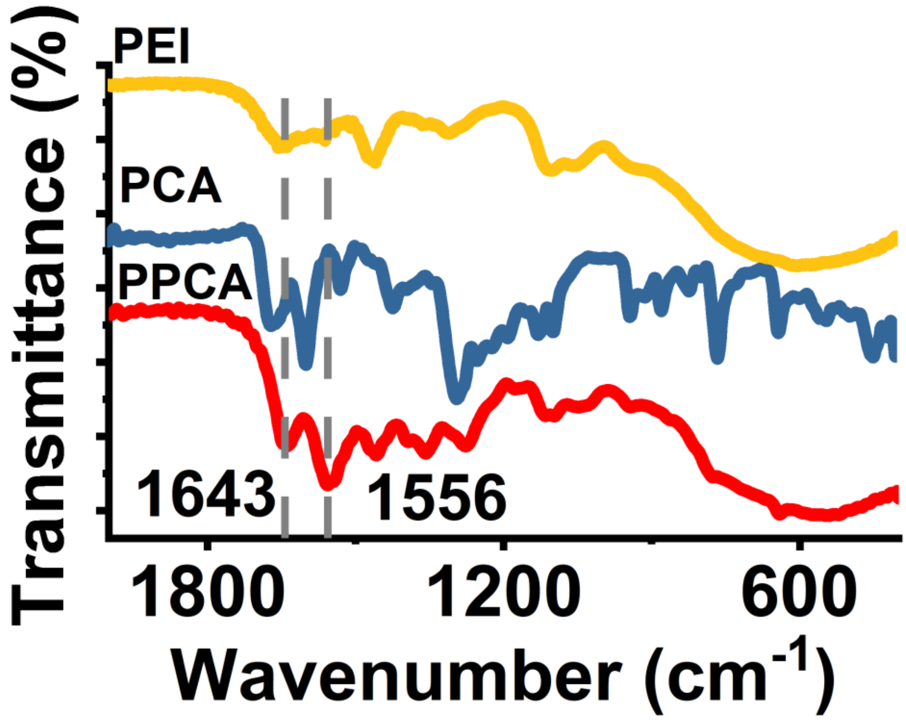

Figure S2. The FTIR results of PEI, PCA and PPCA, showing the synthesis of PPCA polymers.

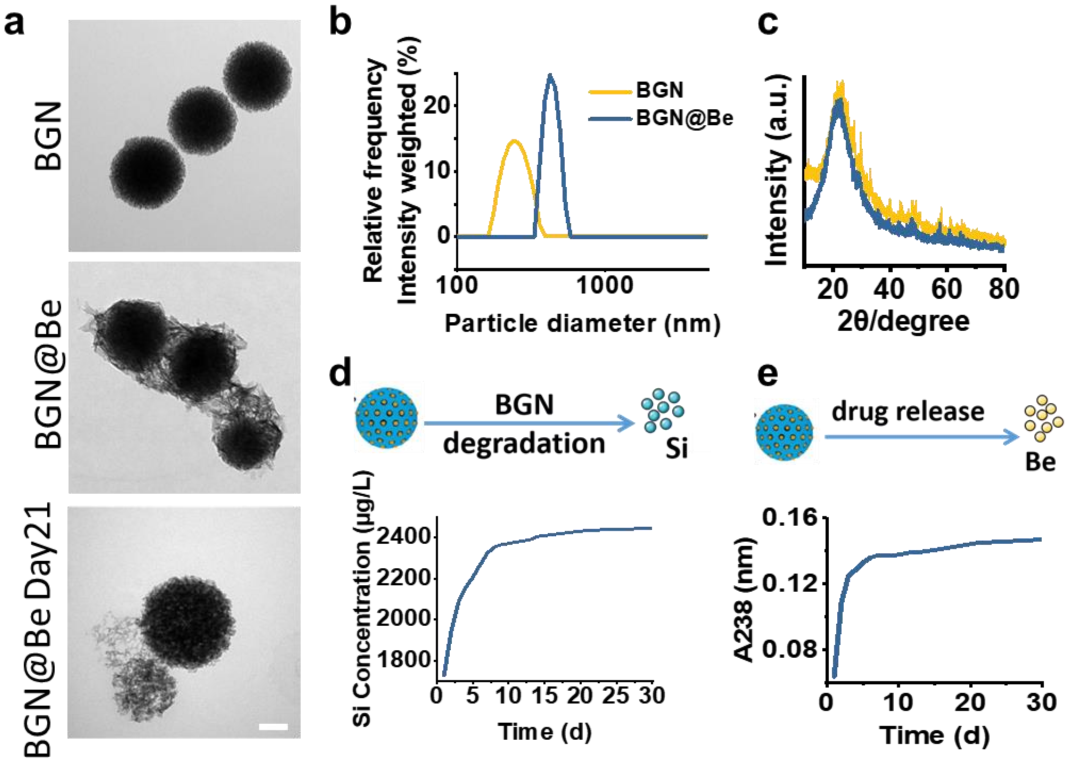

Figure S3. The characterization of BGN and BGN@Be. **a** Representative TEM images of BGN, BGN@Be and the degraded BGN@Be for 21 days. Scale bar = 100 nm. **b** Size distribution and **c** X-ray Diffraction of BGN and BGN@Be. The release of **d** Si element and **e** Be of BGN@Be after soaking in PBS.

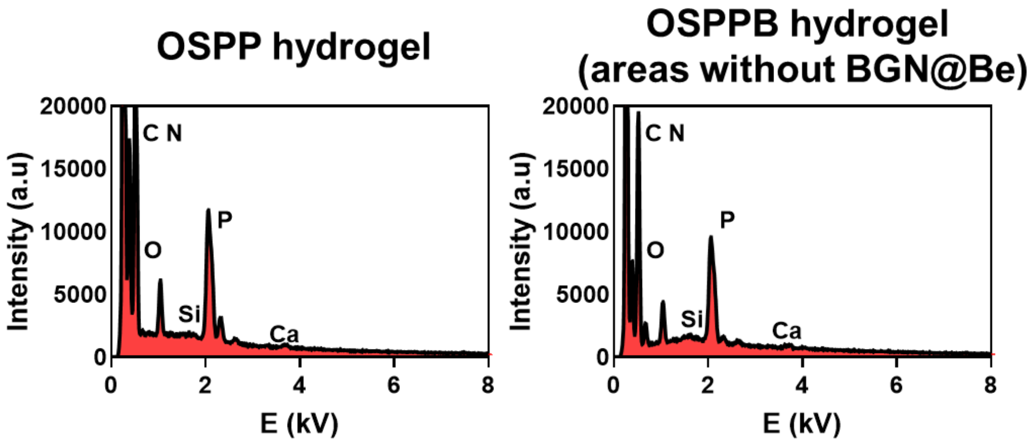

Figure S4. SEM-EDS of OSPP hydrogel and OSPPB hydrogel (areas without BGN@Be distribution).

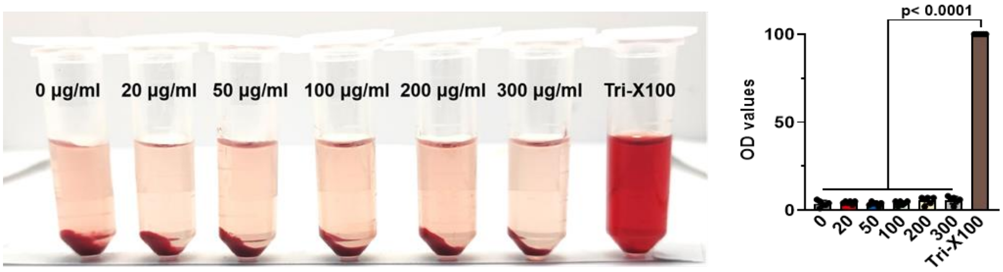

Figure S5. Photographs of RBCs after treatment with OSPPB, with the corresponding statistical results. Data are shown as the means and standard deviations. One-way ANOVA is followed by the Tukey-Kramer method for post hoc multiple comparisons. $n = 6$, $F(6, 35) = 3660$, $p < 0.0001$.

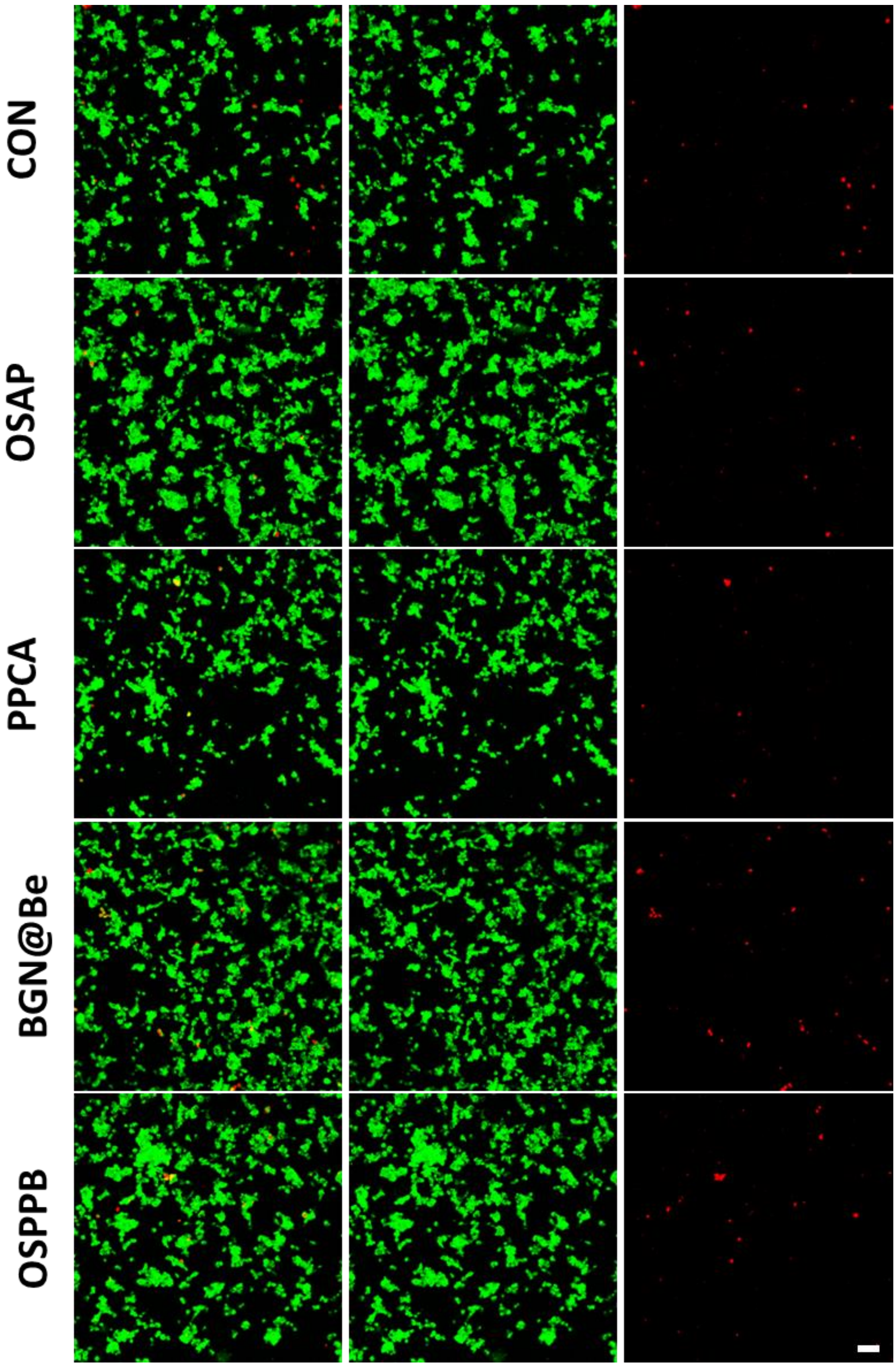

Figure S6. Representative fluorescence images of LIVE/DEAD staining of EPCs after OSPPB treatment. Scale bar = 100 μm.

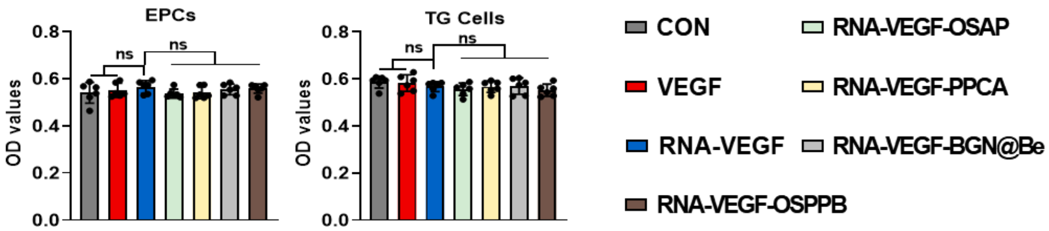

Figure S7. CCK-8 assay of the viability of EPCs and TG cells under the stimulation of different groups. Data are shown as the means and standard deviations. One-way ANOVA is followed by the Tukey-Kramer method for post hoc multiple comparisons. $n = 6$, $F(6, 35) = 0.8479$, $p = 0.5421$ in EPCs. $n = 6$, $F(6, 35) = 1.223$, $p = 0.3181$ in TG cells.

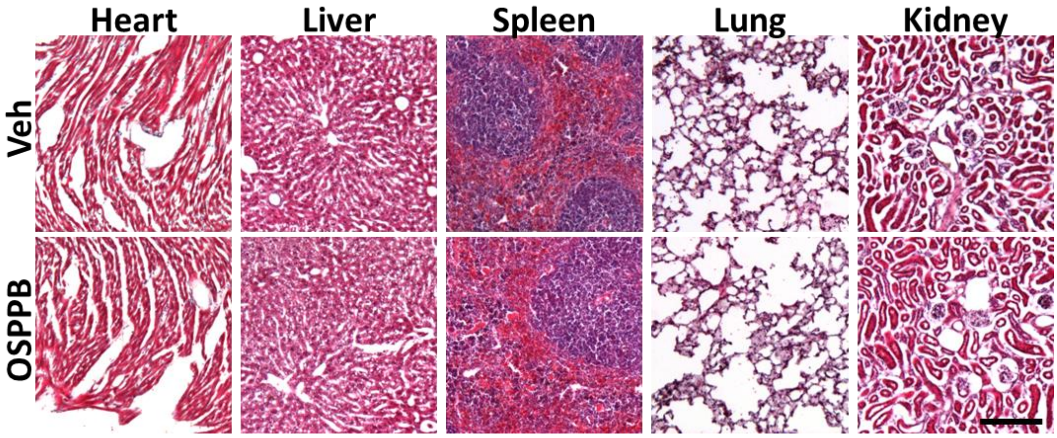

Figure S8. H&E staining of the major organs, including heart, liver, spleen, lungs, and kidneys after OSPPB intraarticular injection. Scale bar = 100 μm.

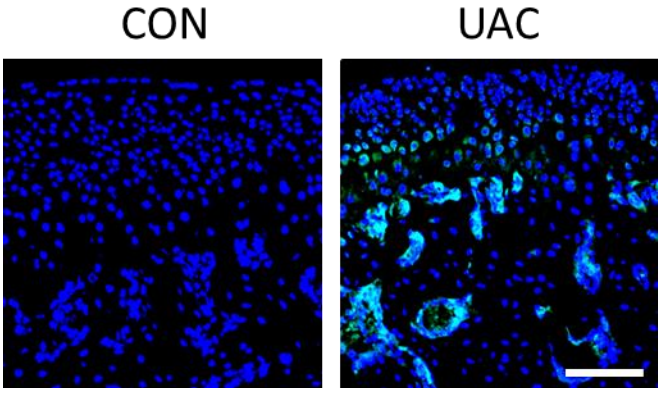

Figure S9. The distribution of the rhodamine B-stained hydrogel in condyles. Scale bar = 100 μm.

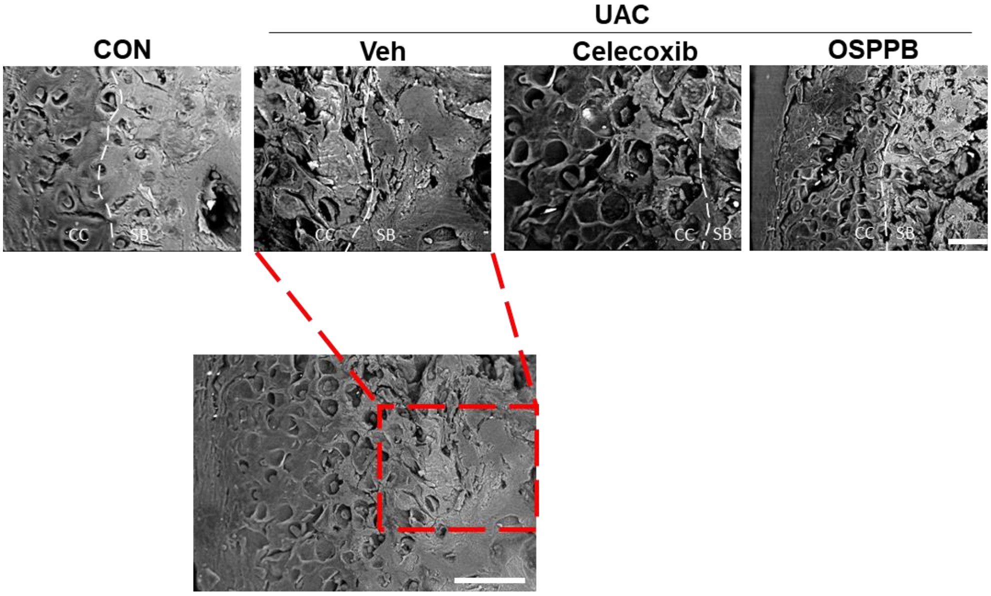

Figure S10. Original images of the condyles used for SEM-EDS. Scale bars = 20 μm (up) and 500 μm (down).

Table S1. Primer sequences used for RT-PCR in the present study.

**Gene name**	**Sequence （5’-3’）**
m-Pdgfb-F	ATGAAATGCTGAGCGACCACT
m-Pdgfb-R	TCAGCCCCATCTTCATCTACGG
m-Ngf-F	ACCACGACTCACACCTTTGTCAAG
m-Ngf-R	CACACACACACAGGCCGTATCTATC
m-Vegf-F	ACATTGGCTCACTTCCAGAAACAC
m-Vegf-R	TGGTTGGAACCGGCATCTTTA
m-Netrin1-F	GTTCGGCGACGAGAACGAA
m-Netrin1-R	TGTGCCTACAGTCACACACCAGA
m-Netrin3-F	AGGTCGGCCAATCGCGT
m-Netrin3-R	CAGGGGCACTGTGAGGGTTAC
m-Netrin4-F	AGATCACCAACCTCCGAGTGC
m-Netrin4-R	GCCCTTGACGATGAAGTCATAGAC
