# Supplementary material for: Neurovascularization inhibiting dual responsive hydrogel for alleviating the progression of osteoarthritis
Source: Nat Commun. 2025 Feb 6;16:1390. doi: 10.1038/s41467-025-56727-8 (PMC11799281; doi:10.1038/s41467-025-56727-8)
Supplement: Supplementary file 2 — Reporting Summary [file 41467_2025_56727_MOESM2_ESM.pdf]

nature portfolio

Corresponding author(s): Kai Jiao
Last updated by author(s): Jan 17, 2025

Reporting Summary

Nature Portfolio wishes to improve the reproducibility of the work that we publish. This form provides structure for consistency and transparency in reporting. For further information on Nature Portfolio policies, see our Editorial Policies and the Editorial Policy Checklist.

Statistics

For all statistical analyses, confirm that the following items are present in the figure legend, table legend, main text, or Methods section.

n/a	Confirmed	
☐	☒	The exact sample size (*n*) for each experimental group/condition, given as a discrete number and unit of measurement
☐	☒	A statement on whether measurements were taken from distinct samples or whether the same sample was measured repeatedly
☐	☒	The statistical test(s) used AND whether they are one- or two-sided<br>*Only common tests should be described solely by name; describe more complex techniques in the Methods section.*
☒	☐	A description of all covariates tested
☐	☒	A description of any assumptions or corrections, such as tests of normality and adjustment for multiple comparisons
☐	☒	A full description of the statistical parameters including central tendency (e.g. means) or other basic estimates (e.g. regression coefficient) AND variation (e.g. standard deviation) or associated estimates of uncertainty (e.g. confidence intervals)
☐	☒	For null hypothesis testing, the test statistic (e.g. F, t, r) with confidence intervals, effect sizes, degrees of freedom and P value noted<br>*Give P values as exact values whenever suitable.*
☒	☐	For Bayesian analysis, information on the choice of priors and Markov chain Monte Carlo settings
☒	☐	For hierarchical and complex designs, identification of the appropriate level for tests and full reporting of outcomes
☒	☐	Estimates of effect sizes (e.g. Cohen's d, Pearson's r), indicating how they were calculated

Our web collection on statistics for biologists contains articles on many of the points above.

Software and code

Policy information about availability of computer code

Data collection	1. For scanning electron microscopy and energy-dispersive X-ray spectroscopy, a field-emission scanning electron microscope (FE-SEM, S-4800, Hitachi, Tokyo, Japan) and Energy-dispersive X-ray spectroscopy (Element EDS System, Ametek, PA, USA) were used.<br>2. The characterization of different components was used ATR-FTIR (FTIR-8400S, Shimadzu, Tokyo, Japan).<br>3. A rheometer (NETZSCH Kinexus Lab+, Bayern, Germany) was used to measure the rheological properties.<br>4. The zeta potential was tested using a Litesizer 500 particle analyzer (Anton Paar, Graz, Austria).<br>5. Fluorescent images were obtained using CLSM (Nikon A1R, Nikon Corporation, Minato ku, Tokyo, Japan)<br>6. Micro-CT scanner (Inveon, Siemens AG, Munich, Germany) was 8. used for bone mass analysis.<br>7. For the AFM test, AFM-based nanoindentation (Keysight 5500, Keysight Technologies, Santa Rosa, CA, USA) was used.<br>8. The electroencephalography activity of S1BF was recorded using an electroencephalography monitoring system (SOLAR3000N, Beijing, China).
Data analysis	1.Image J software v1.48v (National Institute of Health, Bethesda, MD, USA) was used for semi-quantitative analysis.<br>2. Analyses were performed using GraphPad Prism 8.0 (GraphPad Inc., La Jolla, CA, USA).<br>3. Spectral analysis was performed using IR solution software (Shimadzu, Kyoto, Japan)

For manuscripts utilizing custom algorithms or software that are central to the research but not yet described in published literature, software must be made available to editors and reviewers. We strongly encourage code deposition in a community repository (e.g. GitHub). See the Nature Portfolio guidelines for submitting code & software for further information.

Data

Policy information about availability of data

All manuscripts must include a data availability statement. This statement should provide the following information, where applicable:

- Accession codes, unique identifiers, or web links for publicly available datasets
- A description of any restrictions on data availability
- For clinical datasets or third party data, please ensure that the statement adheres to our policy

All data needed to evaluate the conclusions in the paper are present in the paper and/or the Supplementary Materials. Source Data are provided with this paper.

Research involving human participants, their data, or biological material

Policy information about studies with human participants or human data. See also policy information about sex, gender (identity/presentation), and sexual orientation and race, ethnicity and racism.

Reporting on sex and gender	This work does not involve human research
Reporting on race, ethnicity, or other socially relevant groupings	This work does not involve human research
Population characteristics	This work does not involve human research
Recruitment	This work does not involve human research
Ethics oversight	This work does not involve human research

Note that full information on the approval of the study protocol must also be provided in the manuscript.

Field-specific reporting

Please select the one below that is the best fit for your research. If you are not sure, read the appropriate sections before making your selection.

☒ Life sciences ☐ Behavioural & social sciences ☐ Ecological, evolutionary & environmental sciences

For a reference copy of the document with all sections, see nature.com/documents/nr-reporting-summary-flat.pdf

Life sciences study design

All studies must disclose on these points even when the disclosure is negative.

Sample size	A minimum of six independent experiments were carried out for all in vivo and in vitro studies.<br>No sample-size calculation was performed for the in vivo studies. Sample sizes in each experiment were chosen according to practical considerations. The expected effect sizes were large and sufficient for statistical analysis.
Data exclusions	Because no abnormalities were observed, no data were excluded from the study.
Replication	All experiments were performed at least in triplicate and all attempts at replication were successful.
Randomization	Samples and animals were randomly assigned at the time of grouping to minimize any potential bias.
Blinding	The preparation of experimental subjects was blinded from the experimentalist performing the analysis.

Reporting for specific materials, systems and methods

We require information from authors about some types of materials, experimental systems and methods used in many studies. Here, indicate whether each material, system or method listed is relevant to your study. If you are not sure if a list item applies to your research, read the appropriate section before selecting a response.

Materials & experimental systems

n/a	Involved in the study	
[ ]	[x]	Antibodies
[ ]	[x]	Eukaryotic cell lines
[x]	[ ]	Palaeontology and archaeology
[ ]	[x]	Animals and other organisms
[x]	[ ]	Clinical data
[x]	[ ]	Dual use research of concern
[x]	[ ]	Plants

Methods

n/a	Involved in the study	
[x]	[ ]	ChIP-seq
[x]	[ ]	Flow cytometry
[x]	[ ]	MRI-based neuroimaging

Antibodies

Antibodies used	1. β3-tubulin (1:300, ab18207, Abcam, Cambridge, UK)<br>2. protein gene product 9.5 (PGP 9.5; 1:300, ab8189, Abcam)<br>3. CGRP (1:400, 14959, Cell Signaling Technology, Danvers, MA, USA)<br>4. VEGF (1:300, sc-7269, Santa Cruz Biotechnology CA, USA)<br>5. CD31 (1:300, sc-376764, Santa Cruz Biotechnology, Santa Cruz, CA, USA)<br>6. E-cadherin (1:300, ab231303, Abcam)<br>7. cyclooxygenase 2 (COX2; 1:300, sc-376861, Santa Cruz Biotechnology)<br>8. DCC (1:300, sc-515834, Santa Cruz Biotechnology)<br>9. SP (1:300, sc-21715, Santa Cruz Biotechnology).
Validation	All antibodies were acquired from commercial vendors and were validated by the manufacturer for use in the species and assays used in the study.

Eukaryotic cell lines

Policy information about cell lines and Sex and Gender in Research

Cell line source(s)	EPC cells were sourced from Jennio Biotech, Guangdong, China.
Authentication	Cells authenticity were tested by the vendor. We did not perform any additional authentication tests
Mycoplasma contamination	Cell line was tested negative for mycoplasma contamination by the vendor. We did not perform any additional tests.
Commonly misidentified lines<br>(See ICLAC register)	No commonly misidentified cell lines were used.

Animals and other research organisms

Policy information about studies involving animals; ARRIVE guidelines recommended for reporting animal research, and Sex and Gender in Research

Laboratory animals	C57BL/6J mice (8 weeks old, 17–19 g) and thirty Sprague-Dawley rats (1–5 days old) were purchased from the Laboratory Animal Center of the Forth Military Medical University. The mice were kept under specific pathogen-free conditions. The mice were kept in wire stainless cages (five animals per cage) in an animal chamber with a controlled temperature and humidity (24 deg +/– 1 deg and 55% +/– 5%, respectively)
Wild animals	The study did not involve wild animals.
Reporting on sex	Female mice were used in the study.
Field-collected samples	The study did not involve samples collected from the field.
Ethics oversight	All protocols were performed in accordance with the Guidelines for Care and Use of Experimental Animals of the University. The research protocols were approved by the Medical Ethics Committee of the Forth Military Medical University (#20240011).

Note that full information on the approval of the study protocol must also be provided in the manuscript.

Plants

Seed stocks	This work does not involve plants.
Novel plant genotypes	This work does not involve plants.
Authentication	This work does not involve plants.
